# Association between troponin T and ICU mortality, a changing trend

**DOI:** 10.5830/CVJA-2011-034

**Published:** 2012-05

**Authors:** S Hajsadeghi, S Gholami, G Gohardehi, NS Moghadam, AS Sabet, SRJ Kerman, M Moradi, R Mollahoseini

**Affiliations:** Department of Cardiology, Rasoul-e-Akram Hospital, Tehran; University of Medical Sciences, Tehran, Iran; Firoozgar Clinical Research Development Centre, Firoozgar Hospital, Tehran University of Medical Sciences, Tehran, Iran; Medical Student Research Committee, Tehran University of Medical Sciences, Tehran, Iran; Medical Student Research Committee, Tehran University of Medical Sciences, Tehran, Iran; Medical Student Research Committee, Tehran University of Medical Sciences, Tehran, Iran; Medical Student Research Committee, Tehran University of Medical Sciences, Tehran, Iran; Medical Student Research Committee, Tehran University of Medical Sciences, Tehran, Iran; Department of Neurosurgery, Firoozgar Hospital, Tehran; University of Medical Sciences, Tehran, Iran

**Keywords:** troponin T, ICU, mortality, serial measurement, changing trend

## Abstract

**Background:**

Initially elevated levels of troponin predict adverse outcomes in patients admitted to the intensive care unit (ICU). No research team has investigated the changes in concentration of cardiac troponin T (cTnT) during ICU stay and their association with patient outcome.

**Objective:**

We investigated whether the change in cTnT levels during ICU stay could predict outcomes (death or survival).

**Methods:**

In this cohort study, all patients admitted to the medical ICU (10 beds) from January to July 2008 were enrolled. Troponin levels were evaluated within the first 24 hours of ICU admission and on the fourth, seventh and 10th days after admission.

**Results:**

The study population (135 patients) had a mean age of 60.9 ± 21.5 years. The outcome was significantly different with regard to normal or elevated cTnT concentrations on the first and seventh days of follow up (*p* = 0.03 and 0.023, respectively). This difference was non-significant for cTnT levels on the fourth and 10th days after admission (*p* = 0.69 and 0.78, respectively). The change in cTnT levels was not significantly different between the deceased and discharged patients (*p* = 0.4).

**Conclusion:**

Changes in cTnT levels during ICU stay did not show a significant trend (power: 0.26). Patients whose cTnT levels were increased on the first and seventh days of ICU stay had a worse survival, which could be associated with cardiac events on admission or at specific times during the stay in ICU.

## Abstract

Dysfunction of, or injury to the myocardium was recently found to be an important complication in non-cardiac critically ill patients, including those with sepsis, pulmonary embolism, renal insufficiency and acute stroke, resulting in increased concentrations of cardiac troponin T (cTnT).^1^-^5^ The pathophysiology of myocardial injury in critically ill patients is believed to be multifactorial, including the underlying disease process, hypoxaemia and acidosis, as well as therapeutic activities.^6^,^7^

Over the past decade, several studies have indicated that cardiac dysfunction is a frequent and important factor in determining the outcome of critically ill patients.^8^,^9^ Damage to the myocardial cells results in the release of contractile regulating proteins such as cTnT, which is a highly sensitive and specific marker of myocardial injury.^10^,^11^ More importantly, elevated levels of troponin predict a poor prognosis in patients with acute coronary syndromes^12^-^16^ and may also predict adverse outcomes in other patients admitted to the intensive care unit (ICU).

In a medical ICU, patients with elevated levels of troponin T or troponin I admitted without a diagnosis of acute coronary syndrome exhibited a fourfold higher mortality rate.^17^ In patients in a surgical ICU, moderate elevations in troponin I were associated with higher mortality rates, and a longer stay in hospital and in the ICU.^16^-^18^ Some studies^19^,^20^ have shown that increased cTnT levels are associated with increased mortality, but others did not confirm this association.^21^,^22^ The association between elevated troponin level and adverse outcome was uncertain, so the interpretation of elevated troponin levels during critical illness remains unclear.

Cardiac events resulting in elevated biomarkers may occur during ICU stay and have an important effect on patient outcome. There is no research evaluating the relationship between changing levels of any cardiac biomarkers during ICU stay and patients’ outcome. We therefore hypothesised that patients admitted to the ICU with increased cTnT levels were at increased risk of death, and determined whether elevated troponin levels were related to outcome and length of stay and mortality in the ICU. We also investigated whether the change in cTnT levels during ICU stay could predict outcomes (death or survival). Correlation of troponin levels with the most popular disease severity classification system, the Acute Physiology and Chronic Health Evaluation II (APACHE II) score, was also evaluated.

## Methods

In this cohort study, all 135 patients admitted to the medical ICU (10 beds) from January to July 2008 were enrolled consecutively. The patients mostly suffered from sepsis (*n* = 21, 15.5%), stroke (*n* = 11, 8.1%) and pulmonary disease, including chronic obstructive pulmonary disease (COPD) and pneumonia (*n* = 11, 8.1%). This investigation was approved by the ethics committee of the Iran University of Medical Sciences. Verbal consent was obtained from all patients (or from their next of kin) after detailed explanations and a letter of explanation was given.

On admission to the medical ICU, demographic and baseline clinical characteristics, including age, gender and APACHE II score and the diagnoses of all patients were recorded. The APACHE II system incorporates acute physiological variables and chronic health evaluation into a measurement of the risk of in-hospital mortality.^23^ Levels of cardiac biomarkers such as creatine kinase (CK) and creatine kinase isoenzyme (CK-MB), and history of cardiovascular disease were included. All patients in the ICU had electrocardiography (ECG) within 24 hours of admission. The length of stay in the ICU was also recorded. The clinical endpoint was death or discharge from the ICU at any time during hospitalisation.

Troponin measurements were collected within the first 24 hours of ICU admission and on the fourth, seventh and 10th day after admission. This was suggested by the ICU team and was based on their clinical judgment. Troponin T was measured using a radio-immunoassay. The analytical sensitivity (lower detection limit) of this assay is 0.01 μg/l. Troponin concentration of 0.1 μg/l was considered normal. Because of a laboratory mistake, 16 patients did not have troponin T levels measured on admission, but troponins were measured on days four, seven and 10; these data were included in the analysis. Of all patients, 74 patients had the first two, 47 had the first three, and 25 had four serial measurements for cTnT concentrations. Patients did not have cTnT levels measured on discharge or on death.

## Statistical analysis

Baseline characteristics of the negative (normal levels) and positive (elevated levels) cTnT groups were compared using the Pearson’s chi-square test. Continuous variables were compared using the Student’s *t*-test for normally distributed variables, and the Mann–Whitney *U* test if either of these conditions were not met. Linear regression analysis was done to evaluate the independent association between APACHE score and cTnT levels.

For evaluation of the changes in cTnT levels during ICU stay, repeat-measurement ANOVA analysis was carried out. The Breslow method was used to compare patient survival in the Kaplan–Meier analysis. Data are presented as mean ± SD. A *p*-value less than 0.05 was considered significant. Receiver operating characteristics (ROC) were used for the detection of cut-off points for APACHE to predict elevated cTnT levels.

## Results

The study population consisted of 135 patients admitted to the ICU over a period of seven months. There were 73 (54%) men and 62 (46%) women, with a mean age of 60.9 ± 21.5 years, ranging from 15 to 100 years old.

The most common diagnosis was infectious disease, including sepsis (*n* = 21, 15.5%), stroke (*n* = 11, 8.1%), and pulmonary disease, including chronic obstructive pulmonary disease and pneumonia (*n* = 11, 8.1%). There was no significant difference in measured cTnT levels on the first, fourth, seventh and 10th days of ICU stay between patients with different diseases (*p* > 0.05).

History of cardiovascular disease, including MI, hypertension and heart failure was positive in 51 (37%) patients. The frequency of patients with elevated cTnT levels was not significantly different in patients with and without a history of cardiac disease.

On admission, 83 patients (70.3%) had normal cTnT levels, whereas 35 (29.7%) had elevated levels > 0.01 ng/ml, mean = 0.28 ± 0.5 ng/ml (range 0.03–2.00 ng/ml). The clinical and laboratory characteristics of the two groups are shown in Table 1. There was no significant difference between the two groups with normal and elevated cTnT levels in some baseline characteristics, including Glasgow coma score (GCS) (*p* = 0.223) and partial arterial oxygen tension (PaO_2_; *p* = 0.607) on admission. A significant difference was found in the APACHE II score (*p* = 0.003) and serum creatinine levels (*p* = 0.003) between the two groups with different troponin results.

**Table 1:** Baseline Laboratory Parameters Of 118 Critically Ill Patients On Admission To ICU

*Baseline parameters*	*Negative cTnT level (n = 83)*	*Positive cTnT level (n = 35)*	p-*value*
Age (years)	59.8 ± 21.63	61.3 ± 20	0.732
APACHE II score	17.75 ± 7.76	24.3 ± 8.8	0.003
Mean arterial pressure (mmHg)	93.47 ± 13.43	95.17 ± 16.24	0.569
Heart rate (beats/min)	91.56 ± 19	94.74 ± 22.69	0.446
Respiratory rate (per min)	21.41 ± 4.52	20.48 ± 5.7	0.363
PaO_2_ (mmHg)	97.5 ± 56.6	91.2 ± 46.7	0.607
FiO_2_ (%)	61.9 ± 27.5	61 ± 26.4	0.887
HCO_3_ (mmol/l)	25.1 ± 8.34	25.11 ± 12.67	0.995
Sodium (mmol/l)	141 ± 7.05	140.5 ± 6.69	0.713
Potassium (mmol/l)	4.11 ± 0.6	4.11 ± 0.7	0.998
Creatinine (mg/dl)	1.19 ± 0.96	2.58 ± 2.53	0.003
Haematocrit (%)	35.55 ± 9.33	32.5 ± 8.33	0.114
White blood cell count (per ml)	12.22 ± 6.49	12.22 ± 4.97	0.999
pH	7.36 ± 0.09	7.34 ± 0.1	0.422
Systolic blood pressure (mmHg)	127.4 ± 18.6	129.7 ± 18.7	0.545
Diastolic blood pressure (mmHg)	76.49 ± 12.58	77 ± 17.8	0.854
GCS (median)	9	10	0.223
CK (U/l)	236.12 ± 255	420 ± 735	0.262
CK-MB (ng/ml)	28.63 ± 21	128.3 ± 190	0.72
Male/female (ratio)	45/38	17/18	0.531
History of cardiac disease (yes/no)	29/54	12/23	0.959
ST change on ECG (yes/no)	36/47	18/15	0.29

PaO_2_: arterial O_2_ tension, FiO_2_: fraction of inspired oxygen, HCO_3_ : bicarbonate, GCS: Glasgow coma scale, CK: creatine kinase, CK-MB: creatine kinase-MB.

**Fig. 1:**
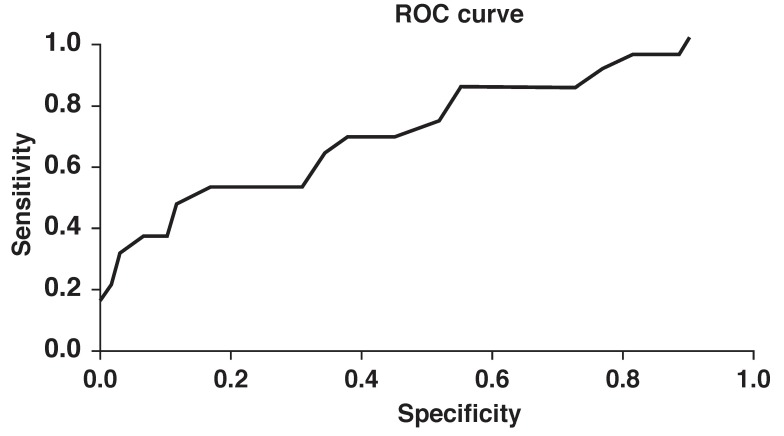
The results of ROC analysis (area under the curve = 0.712 and standard error = 0.072).

Patients with negative baseline troponin T levels had a significantly lower APACHE II score (17.75 ± 7.76 vs 24.3 ± 8.8, *p* = 0.003). The APACHE II score on the fourth day after ICU admission was significantly higher in patients with positive cTnT levels (25.07 ± 8.01 vs 19.06 ± 8.57, *p* = 0.026). According to ROC analysis, an APACHE score of more than 20.5 indicated a positive cTnT with 66.7% sensitivity and 62.1% specificity. An APACHE II score higher than 25.5 indicated a positive cTnT with 50% sensitivity and 83% specificity (*p* = 0.006, area under the curve = 0.712 and standard error = 0.072) (Table 2, Fig. 1).

**Table 2:** The Results Of ROC Analysis

*APACHE score*	*Sensitivity*	*Specificity*
18.5	0.684	0.552
19.5	0.684	0.603
20.5	0.684	0.621
21.5	0.632	0.655
22.5	0.526	0.69
23.5	0.526	0.741
24.5	0.526	0.793
25.5	0.526	0.828
27	0.474	0.879
28.5	0.368	0.897

The italicised rows are suggested as the best points to predict cTnT.

**Fig. 2:**
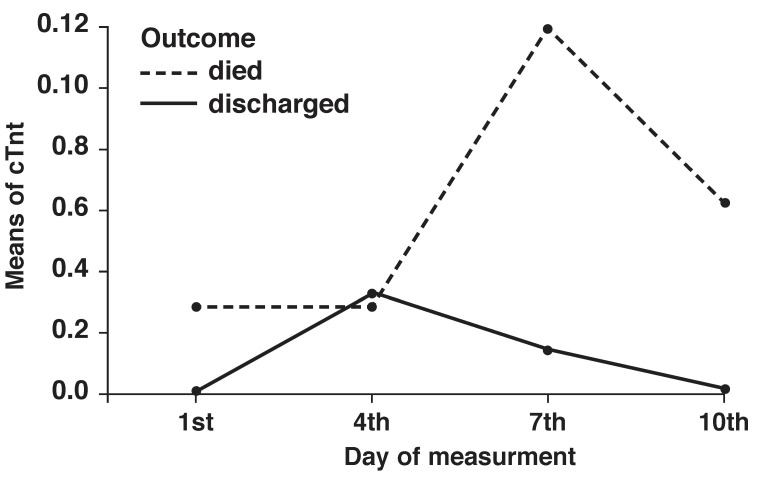
Trend in cTnT levels over four time periods for 25 patients (± SD).

Serial blood sampling for cTnT levels was done in the first 24 hours, and on the fourth, seventh and 10th day after ICU admission (Fig. 2). No significant difference was seen in the changes in troponin levels between deceased and discharged patients (*p* = 0.4). This difference was also non-significant when different diseases were evaluated separately. The difference between troponin levels was not significant between deceased and discharged patients (*p* > 0.05).

The outcome was significantly different with regard to normal or elevated cTnT concentrations on the first and seventh days after admission (Table 3) but there was a non-significant difference between patients with different levels of cTnT on the fourth and 10th days of admission (Table 3). The cTnT concentration was not significantly different between deceased and discharged patients (Table 4).

**Table 3:** Mortality Rate Based On Normal Or Elevated cTnT Concentration In Different Measurements

	*cTnT concentration (μg/l)*	*Deceased n (%)*	p-*value*
cTnT-1 on the 1st day	< 0.1	33 (40)	0.03
	> 0.1	19 (63)	
cTnT-2 on the 4th day	< 0.1	29 (53)	0.69
	> 0.1	11 (58)	
cTnT-3 on the 7th day	< 0.1	18 (44)	0.024
	> 0.1	13 (65)	
cTnT-4 on the 10th day	< 0.1	16 (57)	0.78
	> 0.1	5 (62)	

**Table 4:** cTnT Concentration In Deceased Or Discharged Patients

	*Deceased (μg/l, mean ± SD)*	*Discharged (μg/l , mean ± SD)*	*p-value*
1st day	0.042 ± 0.061	0.03 ± 0.084	0.4
4th day	0.103 ± 0.277	0.028 ± 0.066	0.12
7th day	0.094 ± 0.25	0.852 ± 0.175	0.86
10th day	0.121 ± 0.365	0.053 ± 0.119	0.42

There was a significant association between patients’ outcome and change in cTnT levels in the first and fourth days after admission using chi-square analysis (*p* = 0.029). All patients with a reduced cTnT level died, whereas all patients with an increased cTnT level survived. Also a significant relationship was found between the outcome of patients and the level of cTnT on the fourth and the seventh days of ICU stay (*p* = 0.02). All patients with an elevated cTnT level from the fourth to the seventh day survived, whereas all patients with a decreased cTnT level from the fourth to the seventh day died.

**Fig. 3:**
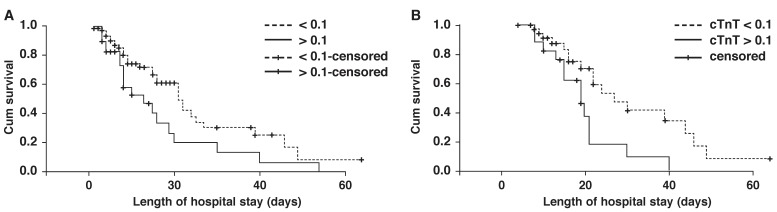
Patient survival and comparison between patients with high and low cTnT levels on (A) first day of admission (*p* = 0.027), (B) seventh day of ICU stay (*p* = 0.012).

The survival rate of patients on the fourth, seventh and 10th days after admission was 87, 74 and 72% in normal patients, and 83, 61 and 56% in patients with elevated cTnT levels, respectively. The estimated mean survival time of patients with elevated cTnT levels on admission was 16.77 (SE = 3.38) days, and it was significantly lower than the group with normal levels on admission, with a mean survival time of 26.08 (SE = 3.15) days (*p* = 0.027). This difference was also significant for cTnT concentration on the seventh day after admission [mean survival 31.6 (SE = 3.6) days in patients with normal cTnT levels vs 19.7 (SE = 2.5) days in patients with elevated cTnT levels, *p* = 0.012] (Fig. 3A, B).

## Discussion

The present study shows that in critically ill patients, elevated cTnT levels were significantly associated with increased mortality, and the survival time was significantly shorter in patients with elevated cTnT than in those with normal cTnT levels.

Cardiac troponin I and troponin T are the most specific and sensitive laboratory markers of myocardial cell injury. However, they may be elevated in patients presenting with many conditions other than acute coronary syndrome.^24^ Elevated levels of cTnT were previously considered a specific marker for the diagnosis of MI. Several recent studies have reported unexpectedly high cTnT levels in the serum of ICU patients who did not have underlying coronary syndrome,^9^,^10^,^25^–^28^ indicating unrecognised myocardial injury during their disease process.

It is reasonable to suggest that critically ill patients are at increased risk of myocardial cell injury. This is due to exposure to many stresses that increase myocardial oxygen demand, whereas the myocardial oxygen supply can be limited by shock, anaemia, tachycardia, hypoxaemia and impaired tissue perfusion.^9^ These events can result in the release of troponin from cardiomyocytes into the serum. In addition, tumour necrosis factor (TNF), produced by inflammatory cells, can depress myocardial function and induce cardiomyocyte apoptosis, which results in low coronary artery flow and decreased ejection fraction, which may lead to necrosis and cTnT release from cardiomyocytes.^29^,^30^ It was found that patients with higher cTnT levels had a shorter survival rate.

The APACHE II scoring system predominately evaluates haemodynamic changes rather than heart function, but cTnT levels could provide a direct marker for cardiac injury, even if clinically unrecognised. In the present study, the APACHE II score on ICU admission was significantly different between patients with elevated and normal cTnT levels. This could have been due to a more serious baseline condition.

Levels of cTnT on admission and on the seventh day could predict an adverse prognosis in critically ill patients, whereas troponin levels on the fourth and 10th days could not. The peak concentration of cTnT was different in these two groups (fourth day in surviving patients and seventh day in patients who died), which in addition to the other findings, represents some undefined importance in the time of assessment of cTnT levels.

Although serial measurements did not provide additional statistical value for risk stratification, the different peaks of troponin levels in deceased and discharged patients are shown in Fig. 2. Different outcomes during ICU stay related to decreasing or increasing cTnT levels have not been reported before. We found that whenever cTnT levels begin to decrease, an adverse outcome could be expected, and increasing cTnT level was a predictor of a favourable outcome. This indicates the significant role that daily changes in cTnT levels play, independent of baseline cTnT levels.

Ammann *et al.*^17^ showed that troponin positivity on admission was associated with a fourfold increased risk of mortality in 58 critically ill patients without acute coronary syndrome. In their study, a significant difference in survival between troponin-positive and troponin-negative patients was found mainly in a subgroup of patients without volume-refractory shock. This implied that the analysis of troponin level could predict mortality in the early but not the late stage of the disease. In another study by Vlad *et al.*, high levels of cTnT on admission had an independent association with in-hospital, short- and long-term mortality in 2 078 patients with acute respiratory distress.^31^

A limitation of the study was that we did not measure renal function in any patient. Renal function is an important factor in cTnT concentrations because the kidney filters cTnT from the blood. An investigation with a larger sample size is also necessary. An inevitable limitation of the sample size was the decreasing number of patients over time because of the death of some between admission and the 10th day. Another limitation was the assay method, which could not detect a cTnT concentration less than 0.01 μg/l, so we did not have exact concentrations for many patients, who were reported to have zero cTnT concentrations.

## Conclusion

We found that levels of cTnT could predict outcome in critically ill patients at specific times. There was a significant association between outcome and cTnT level on the first and seventh days of ICU stay, and a non-significant association with cTnT level on the fourth and 10th days. The level of cTnT had an association with outcome and survival, and was shown to be a predictor of outcome. Cardiac TnT levels during ICU stay did not show a significant trend overall, which may have been due to the small sample size (power: 0.26) but changes in cTnT levels at specific times could be a useful predictor. A study focusing on defining the best time for measurement of cTnT levels would also provide crucial data.
